# Supplementary material for: SMN promotes mitochondrial metabolic maturation during myogenesis by regulating the MYOD-miRNA axis
Source: Life Sci Alliance. 2023 Jan 5;6(3):e202201457. doi: 10.26508/lsa.202201457 (PMC9834662; doi:10.26508/lsa.202201457)
Supplement: Supplementary file 2 [file LSA-2022-01457_SdataFS6.pdf]

Source data for Figure S6H.

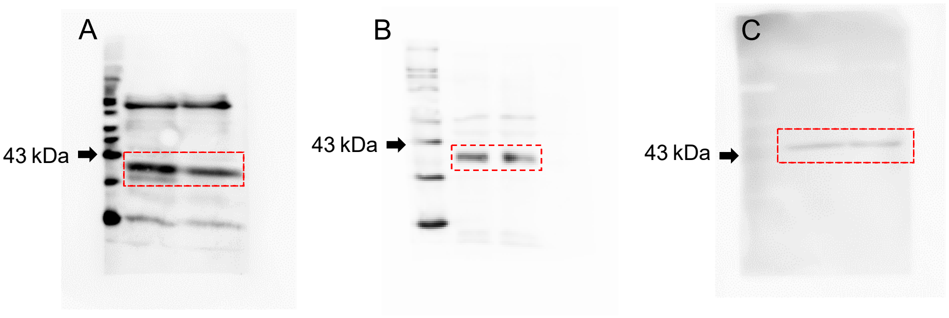

Image A : SMN (38 kDa)   Image B : COX1 (35 kDa)   Image C : β-Actin (45 kDa)

The uncropped gel images of Figure S6H is shown above. The regions surrounded by red square are shown in Figure S6H.
